# Editorial: Options for transition of land towards intensive and sustainable agricultural systems, volume II

**DOI:** 10.3389/fpls.2024.1437911

**Published:** 2024-06-12

**Authors:** Paolo Pesaresi, Evelin Loit

**Affiliations:** ^1^ Department of Biosciences, University of Milan, Milan, Italy; ^2^ Estonian University of Life Sciences, Institute of Agricultural and Environmental Sciences, Tartu, Estonia

**Keywords:** organic farming, soil, manure, crop rotation, crop breeding, ecological corridor

Among the main challenges for the 21^st^ century, the need to feed a global population expected to grow over nine billion by 2050 is of utmost importance. At the same time, the role of agriculture as a source of bioenergy and renewable raw material poses new challenges to the limited croplands, available. Furthermore, a change in paradigm is needed since the increase of food and biomass production must be obtained with a limited environmental impact, by protecting natural resources such as soil and water, safeguarding biodiversity and mitigating the climate change (FAO, 2023).

This Research Topic gathers five contributions highlighting novel strategies and genetic materials to improve agriculture productivity on a sustainable basis. The approaches include Organic Farming, designed to produce high-quality food by keeping at the minimum inputs of resources such as water, energy, fertilizers and pesticides. This is obtained by using fertilizers of organic origin such as animal Manure and by exploiting Crop Rotation to regenerate soil nutrients and microbial diversity (Schröder et al., 2019). Moreover, Artificial ecological corridors, proved to restore ecological environment (Wei et al., 2023), are taken into considerations to understand their long-term impact on restoring soil properties and functions. Finally, the increase of agriculture productivity also requires Novel breeding techniques and genetic materials enabling better adaptation of crops to climate change (Anand et al., 2023).

## Organic farming

In their contribution to this Research Topic, Jiang et al. investigated the feasibility of adopting organic farming practices for the sustainable cultivation of medicinal plants. Data collected over 76 medical plant cultivations in Inner Mongolia revealed that organic agriculture of medicinal plants can improve biodiversity by effectively reducing pesticide and fertilizer use. Moreover, organic farming can help to increase the adaptation of plants to the changing environment, since organically farmed soils have a higher water-holding capacity (Reganold and Wachter, 2016), thus increasing the yield and profits.

## Animal manure

The use of organic amendment accounts for the largest part of soil fertilizing and for enhancing water holding capacity in organic farming (Reichel et al., 2018). Among them compost, animal manure, tree litter and green manure increase soil quality by providing large amounts of fibers and carbon. However, the chemical composition of amendments depends on the different sources and elaboration processes, therefore their quality and application strategy need to be tested before field treatments. In this context, Sæbø et al. explored strategies to manage Nitrogen with solid separated manure, and in particular investigated how the amount of digestate and the Nitrogen: Phosphorous ratio of pig digestate, affected the productivity of Westerwolds ryegrass and red clover in a pot-based experiment. The authors concluded that N-management is required to benefit from the Phosphorus-rich digestate in grass cultivation.

## Crop rotation

In organic farming elaborated crop rotation schemes provide major contributions to regenerate nutrient pools of the topsoil, implement high microbial diversity beneficial for crop production, and prevent erosion and nutrient losses (Schröder et al., 2019). Indeed, a high diversity plant cover is highly sustainable in terms of nutrient use and cycling, compared to monoculture that exhaust soil, requiring higher maintenance costs. In their study, Cui et al. report the results of a field study conducted from 2017–2020 in the Loess Plateau to evaluate the effects of crop rotation sequences on pre-planting and post-harvest soil water storage, annualized crop yield and water use efficiency. The results indicate that the average soil water content was increased after one rotation cycle and that crop rotation had a significant effect on average annual yield and water use efficiency.

## Artificial ecological corridors

United states, Ukraine, China and Argentina together form the natural granary of the world able to guarantee the world food security. These four regions are mainly made of mollisol, a soil type with high organic matter and nutrient-enrich surface. Most of the mollissol area in these regions has been converted to intensive agriculture land during the last century resulting in a tremendous impoverishment of the soil quality in terms of soil properties, such as soil organic carbon, structure, pH, cation exchange capacity and of degradative processes such as erosion and nutrient depletion affecting crop yields (Xu et al., 2020). For instance, land degradation due to cultivation has reduced crop yields by 20–40% in North America. Therefore, strategies to restore the quality of mollisols in these high productive regions are needed. In their contribution, Xu et al. explored the impact of artificial ecological corridors on the recovery process of mollisols in China, revealing the great application potential of this low-cost soil recovery strategy.

## Novel crops that better adapt to climate change

From the 1960s through the 1990s, yields of rice and wheat doubled thanks to the breeding programs developed in the frame of the green revolution. The new, high-yielding wheat varieties developed by Norman Borlaug at the research station at Campo Atizapan in Mexico were characterized by short stem and disease resistance and gave a major contribution to fight hunger in the world (Rajaram, 2011). To keep doing that between now and 2050, we’ll need another green revolution to increase crop productivity in the context of climate change. This will also require novel breeding tools and the possibility to test the new genetic materials in locations characterized by abiotic stress conditions, such as drought, as reported in the contribution of Behera et al., where the performance of 95 diverse sorghum genotypes was evaluated under semi-arid conditions using a multi-trait-based selection approach.

## Author contributions

PP: Writing – original draft, Writing – review & editing. EL: Writing – review & editing.
